# Supplementary material for: Resolving the full spectrum of human genome variation using Linked-Reads
Source: Genome Res. 2019 Apr;29(4):635–45. doi: 10.1101/gr.234443.118 (PMC6442396; doi:10.1101/gr.234443.118)
Supplement: Supplemental Material [file supp_gr.234443.118_Supplemental_longranger-2.2.zip › longranger-2.2.tar.bz2!/lr2.2-public/lib/stan/cmdstan/stan/doc/api/html/expl__leapfrog_8hpp_source.html]

Stan: src/stan/mcmc/hmc/integrators/expl\_leapfrog.hpp Source File


|  |  |
| --- | --- |
|  | Stan  2.5.0  probability, sampling & optimization |


- Main Page
- Related Pages
- Namespaces
- Classes
- Files

- File List
- File Members

All Classes Namespaces Files Functions Variables Typedefs Enumerations Enumerator Friends Macros Pages

- src
- stan
- mcmc
- hmc
- integrators

expl\_leapfrog.hpp

Go to the documentation of this file.

1 #ifndef STAN\_\_MCMC\_\_EXPL\_\_LEAPFROG\_\_BETA

2 #define STAN\_\_MCMC\_\_EXPL\_\_LEAPFROG\_\_BETA

3

4 #include <stan/math/matrix/Eigen.hpp>

5 #include <stan/mcmc/hmc/integrators/base\_leapfrog.hpp>

6

7 namespace stan {

8

9  namespace mcmc {

10

11  template <typename H, typename P>

12  class expl\_leapfrog: public base\_leapfrog<H, P> {

13

14  public:

15

16  expl\_leapfrog(std::ostream\* o=0): base\_leapfrog<H, P>(o) {};

17

18  void begin\_update\_p(P& z, H& hamiltonian, double epsilon) {

19  z.p -= epsilon \* hamiltonian.dphi\_dq(z);

20  }

21

22  void update\_q(P& z, H& hamiltonian, double epsilon) {

23  Eigen::Map<Eigen::VectorXd> q(&(z.q[0]), z.q.size());

24  q += epsilon \* hamiltonian.dtau\_dp(z);

25  }

26

27  void end\_update\_p(P& z, H& hamiltonian, double epsilon) {

28  z.p -= epsilon \* hamiltonian.dphi\_dq(z);

29  }

30

31  };

32

33  } // mcmc

34

35 } // stan

36

37

38 #endif

stan::mcmc::expl\_leapfrog::update\_q

void update\_q(P &z, H &hamiltonian, double epsilon)

**Definition:** expl\_leapfrog.hpp:22

stan::mcmc::expl\_leapfrog::expl\_leapfrog

expl\_leapfrog(std::ostream \*o=0)

**Definition:** expl\_leapfrog.hpp:16

stan::mcmc::expl\_leapfrog::begin\_update\_p

void begin\_update\_p(P &z, H &hamiltonian, double epsilon)

**Definition:** expl\_leapfrog.hpp:18

base\_leapfrog.hpp

stan::mcmc::expl\_leapfrog

**Definition:** expl\_leapfrog.hpp:12

Eigen.hpp

stan::mcmc::expl\_leapfrog::end\_update\_p

void end\_update\_p(P &z, H &hamiltonian, double epsilon)

**Definition:** expl\_leapfrog.hpp:27

stan::mcmc::base\_leapfrog

**Definition:** base\_leapfrog.hpp:13


---

[ Stan Home Page ]

*© 2011–2014,
Stan Development Team.*
